# Supplementary material for: Mental health symptoms in a cohort of hospital healthcare workers following the first peak of the COVID-19 pandemic in the UK
Source: BJPsych Open. 2020 Dec 29;7(1):e24. doi: 10.1192/bjo.2020.150 (PMC7844153; doi:10.1192/bjo.2020.150)
Supplement: Supplementary file 1 [file S2056472420001507sup001.zip › S2056472420001507sup002.docx]

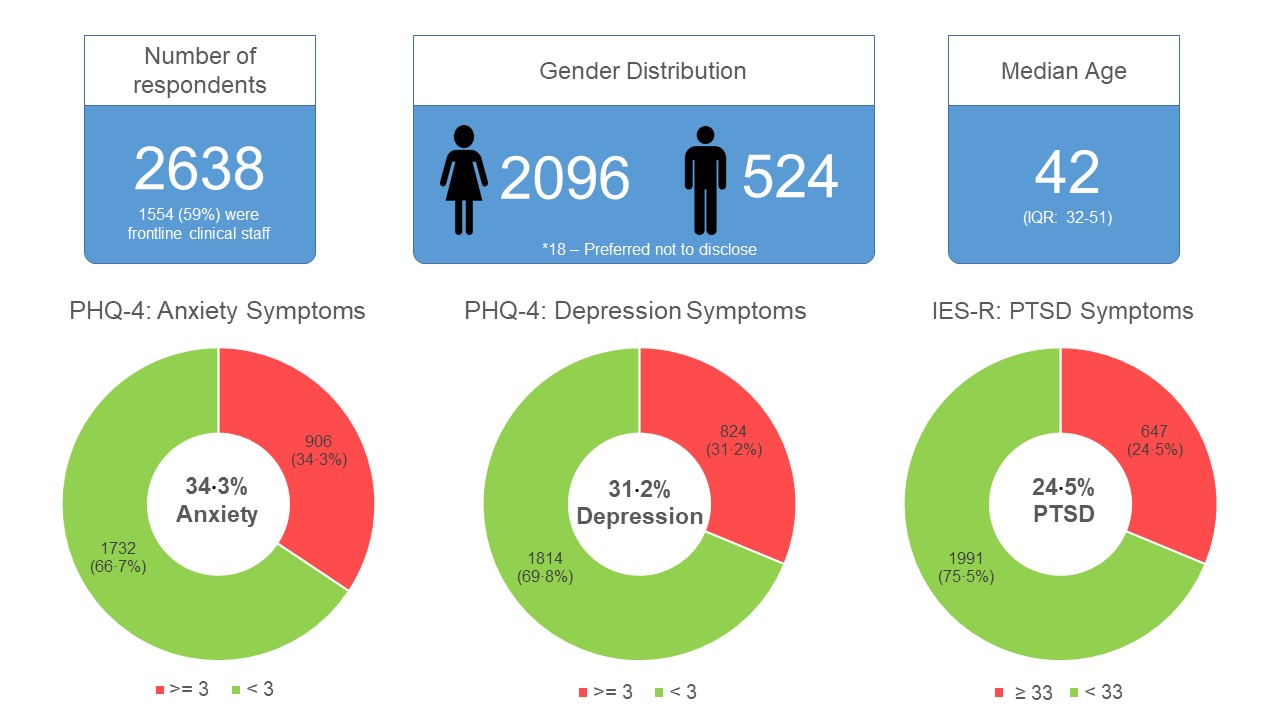


**Supplementary Fig. 1:** A summary of the key demographic data of survey participants, PHQ-4 and IES-R scores
