# Supplementary material for: Mental health symptoms in a cohort of hospital healthcare workers following the first peak of the COVID-19 pandemic in the UK
Source: BJPsych Open. 2020 Dec 29;7(1):e24. doi: 10.1192/bjo.2020.150 (PMC7844153; doi:10.1192/bjo.2020.150)
Supplement: Supplementary file 1 [file S2056472420001507sup001.zip › S2056472420001507sup003.docx]

**PHQ-4: Anxiety Subscale Score >= 3**

**Odds Ratio (95% CI)**


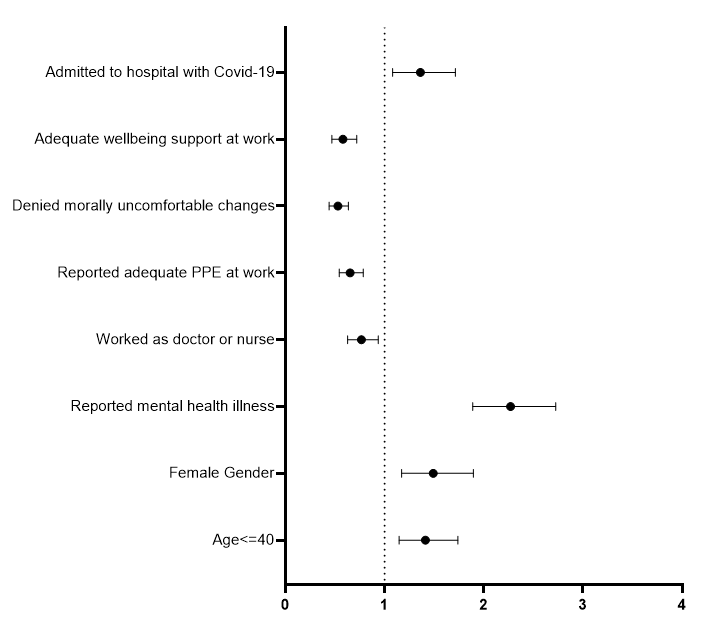


|  |  |
| --- | --- |
| Admitted to hospital with Covid-19 | 1·4 (1·1-1·7); p*=*0·009 |
|  |  |
|  |  |
| Wellbeing support available at work | 0·6 (0·5-0·7); p*<*0·001 |
| Denied morally uncomfortable changes | 0·5 (0·4 – 0·6); p*<*0·001 |
| Reported adequate PPE at work | 0·7 (0·5 – 0·8); p*<*0·001 |
| Worked as a doctor or nurse | 0·8 (0·6-0·9); p*=*0·009 |
| A history of mental health conditions | 2·3 (1·9 – 2·7); p*<*0·001 |
| Female gender | 1·5 (1·2 – 1·9); p*=*0·001 |
| Age <= 40 | 1·4 (1·1 – 1·7); p*=*0·001 |

**Odds Ratio (95% CI)**

**Supplementary Figure 2:** Factors associated with clinically significant anxiety symptoms in adjusted analysis
